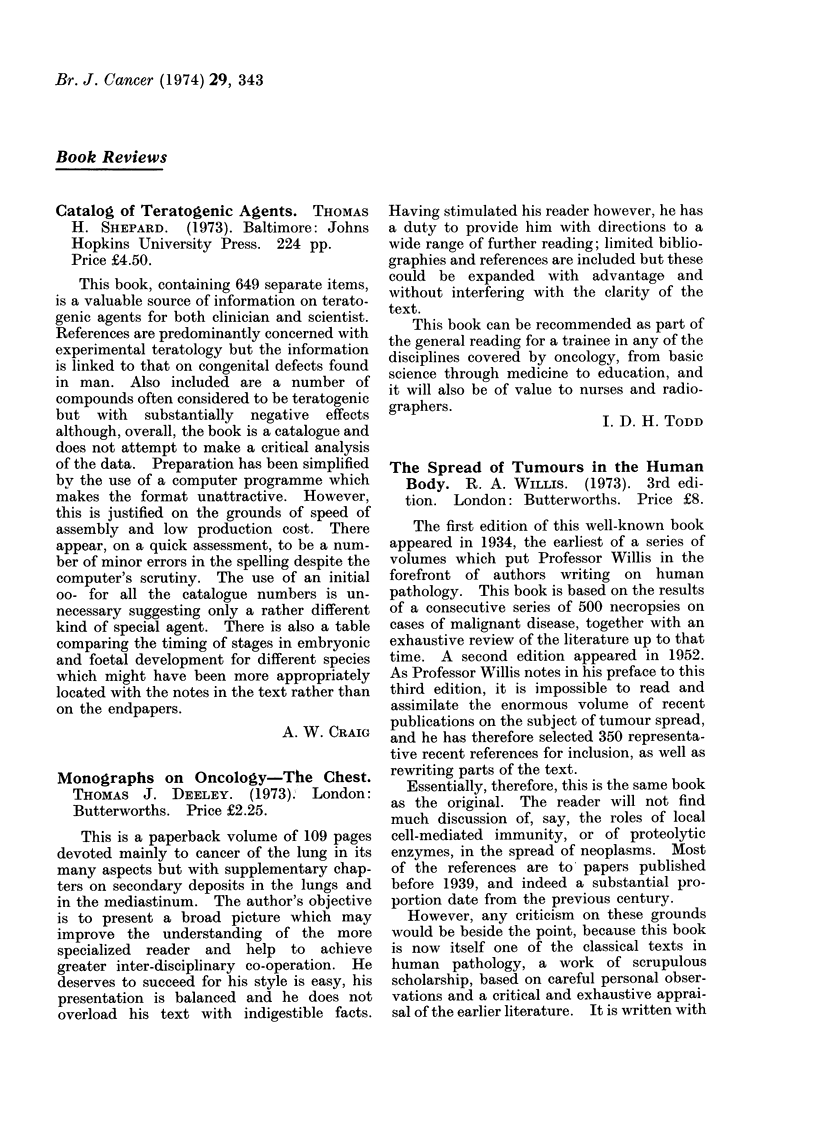# Monographs on Oncology—The Chest

**Published:** 1974-04

**Authors:** I. D. H. Todd


**Monographs on Oncology—The Chest.** THOMAS J. DEELEY. (1973). London: Butterworths. Price £2.25.

This is a paperback volume of 109 pages devoted mainly to cancer of the lung in its many aspects but with supplementary chapters on secondary deposits in the lungs and in the mediastinum. The author's objective is to present a broad picture which may improve the understanding of the more specialized reader and help to achieve greater inter-disciplinary co-operation. He deserves to succeed for his style is easy, his presentation is balanced and he does not overload his text with indigestible facts. Having stimulated his reader however, he has a duty to provide him with directions to a wide range of further reading; limited bibliographies and references are included but these could be expanded with advantage and without interfering with the clarity of the text.

This book can be recommended as part of the general reading for a trainee in any of the disciplines covered by oncology, from basic science through medicine to education, and it will also be of value to nurses and radiographers.

I. D. H. TODD